# Semi-supervised synthesis of 7T MRI from 3T using 3D FR-U-Net with anatomical segmentation consistency assessment

**DOI:** 10.1371/journal.pone.0333499

**Published:** 2025-11-06

**Authors:** Richard Acs, Hanqi Zhuang

**Affiliations:** College of Engineering and Computer Science, Florida Atlantic University, Boca Raton, Florida, United States of America; University of Bergamo: Universita degli Studi di Bergamo, ITALY

## Abstract

Ultra-high-field 7T MRI provides substantial benefits for neuroimaging, including improved resolution and contrast, but remains limited by high costs and restricted accessibility. In this study, we propose a semi-supervised 3D multi-scale fusion residual U-Net (semi supervised 3D FR-U-Net) to synthesize 7T MRI volumes from 3T input using a patch-based architecture optimized for low-data settings. In addition to evaluating conventional synthesis metrics such as PSNR, SSIM, and NMSE, we introduce a novel segmentation-based assessment using the VolBrain pipeline to quantify anatomical fidelity. Our model outperforms prior methods—even without preprocessing steps like skull stripping—and achieves high fidelity in global brain morphology and basal ganglia structures. However, significant asymmetry errors in hippocampal segmentation highlight limitations in preserving fine, clinically critical anatomy. To address the disconnect between technical performance and clinical applicability, we emphasize the use of interpretable, segmentation-derived metrics to bridge the gap between research advances in synthetic MRI and real-world diagnostic relevance. These findings underscore the importance of region-specific evaluation and demonstrate how structural metrics can guide the real-world applicability of synthetic MRI, particularly when expert radiological review is not feasible.

## Introduction

Magnetic resonance imaging (MRI) is one of the most widely used medical imaging modalities, offering high-resolution, non-invasive visualization of soft tissues. Unlike computed tomography (CT) or X-ray imaging, which rely on ionizing radiation, MRI leverages strong magnetic fields and radiofrequency pulses to generate detailed anatomical and functional images [1]. This versatility has made MRI an essential tool in various clinical and research applications, including neurology, oncology, cardiology, and musculoskeletal imaging [2–4]. The quality and diagnostic power of an MRI scan are influenced by several factors, one of the most critical being the strength of the magnetic field, measured in tesla (T) [5].

Higher-field MRI scanners, such as ultra-high-field 7T systems, provide substantial advantages over standard 3T and 1.5T MRIs, offering improved signal-to-noise ratio (SNR), enhanced tissue contrast, and greater spatial resolution [5]. These benefits are particularly significant in neuroimaging, where 7T MRI enables the visualization of fine-scale structures such as cortical layers, hippocampal subfields, and small subcortical nuclei, supporting more precise structural analysis and improved lesion detection [6]. As a result, 7T has become an important tool for selected clinical and research applications—especially in the study of neurodegenerative diseases, epilepsy, and multiple sclerosis [7,8]. However, 7T imaging also introduces significant technical challenges, including B0 and B1 field inhomogeneities, increased susceptibility to artifacts, and higher specific absorption rate (SAR), which can limit performance in certain sequences [6]. For instance, while 7T enhances contrast in susceptibility-weighted and T2*-weighted imaging, it may produce suboptimal results in sequences like T1-weighted MPRAGE without careful protocol optimization. Therefore, 7T does not consistently outperform 3T across all imaging contexts, and its advantages depend heavily on the imaging task, protocol, and anatomical target.

In addition to these technical considerations, the widespread adoption of 7T remains constrained by high cost, limited availability, and logistical barriers [5]. This makes it a valuable but resource-intensive modality, best reserved for cases where its benefits clearly outweigh these constraints. In many routine or large-scale imaging scenarios—particularly when the expected diagnostic gain is marginal—acquiring a full 7T scan may not be justified. In such contexts, algorithmic synthesis of 7T-equivalent images from more accessible 3T scans presents a promising alternative.

Given these challenges, there is strong motivation to develop deep learning-based approaches for synthesizing high-field MRI from lower-field acquisitions, particularly for translating 3T MRI into 7T-equivalent images. By generating high-quality 7T-like scans from more widely available 3T data, such approaches have the potential to enhance clinical imaging, improve diagnostic precision, and expand the utility of ultra-high-field imaging without requiring direct access to 7T scanners [9]. Additionally, the ability to synthesize 7T images from large-scale 3T datasets can facilitate augmentation of existing 7T data, enabling the development of more robust machine learning models for disease classification, segmentation, and other downstream tasks [9].

Recent advances in deep learning, particularly generative models such as generative adversarial networks (GANs), diffusion models, and transformer models, have demonstrated promise in medical image synthesis [9]. Prior studies on high-field MRI synthesis have explored various methodologies, including slice-by-slice synthesis, full-volume synthesis, and hybrid patch-based approaches [9–12]. While slice-wise synthesis methods are computationally efficient and require less memory, they often introduce inconsistencies between adjacent slices, leading to volumetric discontinuities that degrade anatomical fidelity [9]. Full-volume synthesis methods, on the other hand, provide more structurally coherent reconstructions by accounting for inter-slice dependencies, but they typically require larger datasets and substantial computational resources [9].

A major limitation in the field of 7T MRI synthesis is the scarcity of publicly available datasets. Unlike 3T and 1.5T MRI, for which numerous large-scale datasets exist, high-field (≥7T) MRI datasets are limited, with most being private or containing only a small number of subjects [13]. This lack of data presents a significant challenge for training deep learning models, as large datasets are often necessary to achieve generalizable and high-fidelity synthesis. Currently, there are an estimated 100 7T scanners in use, making the development of new datasets mostly limited to small single-institutional cohorts [14]. In this study, we aim to explore volumetric synthesis methods using a recently curated dataset of 10 paired 3T and 7T T1-weighted MRI volumes [15]. More specifically, we present a semi-supervised 3D multi-scale fusion residual U-Net (semi-supervised 3D FR-U-Net) to synthesize full 7T MRI volumes from input 3T volumes. While U-Net architectures have been widely used in MRI synthesis [9], recent advances in diffusion models (DDPMs), vision transformers, and GANs have set new benchmarks in image synthesis quality. However, these models often require large-scale datasets and extensive computational resources, making them impractical for low-resource settings. Our approach retains the computational and data efficiency of U-Nets while integrating residual block structures and multiscale fusion—techniques inspired by high performance segmentation models and vision transformers—to improve representational capacity and cross-scale consistency in MRI synthesis. Additionally, we leverage unpaired 7T data through semi-supervised learning to enhance anatomical fidelity of synthetic high-field MRI structure. Previous approaches to MRI synthesis have used semi-supervised learning [16,17]. However, our study is the first to merge semi-supervised learning into a 3D-patch based U-net approach. Thus, we demonstrate a scalable and efficient alternative for MRI synthesis when paired data is limited, a common challenge in real-world clinical applications of ultra-high-field (7T) MRIs.

This work presents several key contributions to the field of ultra-high-field MRI synthesis under small-data constraints. We utilize a recently curated public dataset consisting of only 10 paired 3T and 7T T1-weighted MRI volumes, making this one of the smallest-scale studies to date to perform direct full-volume synthesis rather than conventional slice-wise approaches. While prior studies on this dataset have focused exclusively on 2D synthesis methods [17,18], and Cui *et al*. applied 3D patches only after skull stripping and harmonization for microbleed synthesis [14], our study is the first to conduct full-volumetric synthesis from minimally preprocessed 3T data. To maximize data efficiency and generalization, we adopt a 3D patch-based U-Net architecture and further enhance anatomical fidelity through a semi-supervised learning framework that leverages unpaired 7T scans for domain adaptation.

In addition to this novel synthesis pipeline, we introduce an anatomical segmentation evaluation framework based on VolBrain, an automated brain segmentation tool built on a large ensemble of CNNs [19]. Beyond standard Dice scores, we propose segmentation consistency derived metrics that capture region-specific anatomical accuracy and asymmetry. This structure-based analysis provides valuable insights into clinical utility without requiring manual annotation when radiologist review is unavailable. Our contributions are summarized as follows:

1. Demonstrating the feasibility of volumetric ultra-high-field MRI synthesis with extremely small datasets (10 paired scans).
2. Presenting a novel semi-supervised 3D multi-scale fusion residual U-Net (semi-supervised 3D FR-U-Net).
3. Enhancing anatomical fidelity through semi-supervised learning, leveraging unpaired data for improved generalization.
4. Utilizing segmentation-based volumetric fidelity metrics to provide interpretable, anatomically-relevant evaluations beyond conventional pixel-level metrics.

### Related approaches

The task of synthesizing 7T MRI from 3T images is best characterized as a cross-domain image translation problem rather than a traditional super-resolution task. While 7T MRI often provides higher spatial resolution, the more critical challenge lies in modeling the differences in contrast, signal properties, and anatomical representation between the two field strengths. Early approaches relied on non-deep learning techniques. For instance, Bahrami *et al*. [20] employed a random forest-based regression model to map 3T images to 7T, while another study utilized a canonical correlation analysis (CCA)-based approach for synthesis [21]. Although these methods established initial benchmarks, they struggled to preserve structural similarity and anatomical fidelity, limiting their clinical applicability.

With the rise of deep learning, CNN-based and generative models have become the dominant methods for high-field MRI synthesis. Bahrami *et al*. [22] introduced a deep CNN-based approach, which outperformed previous traditional techniques but suffered from limited generalization due to small dataset sizes. More advanced generative models have since been applied to high-field MRI synthesis. Wu *et al*. demonstrated that diffusion probabilistic models (DDPMs) can achieve high structural similarity and anatomical fidelity while reducing image distortion [23]. Similarly, conditional GANs have been used to synthesize 7T images from 3T counterparts, achieving improvements in image quality, artifact suppression, sharpness, and contrast, as assessed through manual radiological Likert scale evaluations [24].

However, these deep learning approaches typically require large datasets, which pose a significant challenge given the scarcity of 7T scanners. For example, the DDPM method used 928 MRI subjects to generate a paired low-high resolution dataset [23], while a cycle GAN-based method relied on a privately curated dataset of 122 paired 3T and 7T MRI volumes [24]. Through recent literature, most paired 3T-7T studies have been conducted using as few as 15 patient scans, with larger studies restricted to private datasets, limiting model generalization [13,25].

To address these data limitations, researchers have developed techniques for improving deep learning model performance with small datasets. One approach involved integrating wavelet-based affine transformation layers, achieving high structural similarity and PSNR performance on just 15 paired MRI volumes [25]. Another strategy, Unlimited Data Augmentation (UDA), leveraged deformable registration to artificially expand a 15-pair dataset, integrating a cycle GAN for 7T synthesis [13]. Additionally, various deep learning architectures have been explored for small datasets, including V-Net-based models [14], cycle GAN approaches [18], and 3D anisotropic U-Net models for 1.5 to 3T synthesis [26,27]. Another study employed a wavelet-based frequency attention network with semi-supervised dual-domain consistency learning to synthesize 7T slices from 3T inputs [17].

Most existing studies focus on 2D slice-based synthesis, which has lower data requirements but sacrifices anatomical consistency across slices. In contrast, 3D synthesis improves anatomical coherence but demands significantly larger datasets. Of the literature reviewed, two studies utilized volumetric inputs and outputs, a V-Net-based 3D patch approach [14], and 3D patch-based U-Net approach [28]. Such studies are typically accompanied by dice scores against a segmentation ground truth to evaluate their volumetric fidelity in specific structural areas, such as hippocampal subfields [28]. In a recent 2D-based approach, Eidex *et al*. [29] introduced the 7T-Restormer, an efficient transformer-based model capable of synthesizing quantitative 7T relaxometry maps from combined 1.5T and 3T inputs, showing improved generalization across field strengths. However, their transformer based approach, even when focused on efficiency, required over 100 training MRI volumes,typical of state of the art architecture. Thus, U-Net variations and GAN-based models with data augmentation strategies remain dominant in small-sample MRI synthesis studies, both on 2D and 3D data.

In summary, prior work is often limited by dependence on large or private datasets, the use of predominantly 2D architectures that introduce inter-slice discontinuities, and the absence of region-specific anatomical evaluation beyond global similarity metrics. Many also require extensive preprocessing (e.g., skull stripping), which can limit real-world applicability. This study addresses these issues by enabling volumetric synthesis in a low-data setting, leveraging unpaired 7T data to improve generalization, minimizing preprocessing, and incorporating segmentation-based metrics for anatomically relevant assessment.

## Materials and methods

This study investigates the synthesis of 7T MRI volumes from 3T inputs through several GAN and U-net based approaches. In these methods, either individual 2D slices or 3D patches are synthesized and subsequently assembled to reconstruct the final volumetric image. The quality of the synthesized volumes is assessed through both quantitative metrics (PSNR, SSIM, NMSE) and segmentation-based volumetric analysis. All experiments were conducted on an NVIDIA RTX A6000 GPU (48 GB memory), 8-core CPU, and 45 GB system RAM, running Python 3.13.5 with TensorFlow 2.16.1, CUDA 12.2, and cuDNN 8.9 on Ubuntu 22.04.

All data were preprocessed following the protocol in Sect ‘Data pre-processing’, ensuring consistency across training and evaluation. We then trained and compared three models, a 3D multi-scale fusion residual U-Net (3D FR-U-Net), a semi-supervised 3D multi-scale fusion residual U-Net (semi-supervised 3D FR-U-Net), and a 2D Slice-by-Slice Pix2Pix Baseline. An ablation study was also conducted to demonstrate the effectiveness of the components of our semi-supervised 3D FR-U-Net.

Each model was evaluated using 10-fold cross-validation, and performance was measured using PSNR, SSIM, and NMSE, as detailed in ’Quantitative Evaluation Metrics’. Additionally, we conducted segmentation analysis on the best-performing model to assess structural consistency, as described in ’Volumetric Evaluation Metrics’. The following sections provide a detailed breakdown of the dataset, model architectures, and evaluation methodology.

### Datasets

In this study, we utilize the 7T and 3T Paired Brain MRI Dataset published by Chen *et al*. [15], which consists of paired MRI scans acquired at both 3T and 7T field strengths. The dataset comprises 10 subjects, each with a 3T and a 7T T1-weighted and T2-weighted MRI volume for a total of 4 volumes per subject. The 3T scans were acquired using a Siemens MAGNETOM Prisma 3T scanner, while the 7T scans were obtained using a Siemens MAGNETOM Terra 7T scanner. Both scans were acquired with high isotropic resolution and underwent preprocessing to ensure alignment between the 3T and 7T images. The dataset includes co-registered images, allowing for voxel-wise comparisons between the real and synthesized 7T images. The 7T T1-weighted MP2RAGE sequence acquires two images at different inversion times, each using a separate flip angle (FA1 and FA2), which are then combined to produce a single high-contrast, bias-corrected T1-weighted image. These flip angles are not alternated during acquisition but correspond to distinct readouts that enhance T1 contrast and reduce B1+ inhomogeneity effects, which is particularly important at 7T field strength [6]. Table 1 shows key acquisition characteristics of the different scan types.

**Table 1:** Acquisition characteristics of 7T and 3T MRI images. All scans were acquired using the MPRAGE protocol.

Scan Type	MRI Volume Dimensions	Resolution	TR, TE, FA
3T T1w	208 × 320 × 320	0.8 × 0.8 × 0.8 mm^3^	TR = 2,400 ms, TE = 2.2 ms, FA = 8^°^
7T T1w	256 × 304 × 308	0.65 × 0.65 × 0.65 mm^3^	TR = 6,000 ms, TE = 1.91 ms, FA1 = 4^°^, FA2 = 4^°^

(S × C × T), S = Sagittal, C = Coronal, T = Transverse. Acquisition characteristics include volume dimensions, resolution, repetition time (TR), echo time (TE), and flip angle (FA).

**Fig 1:**
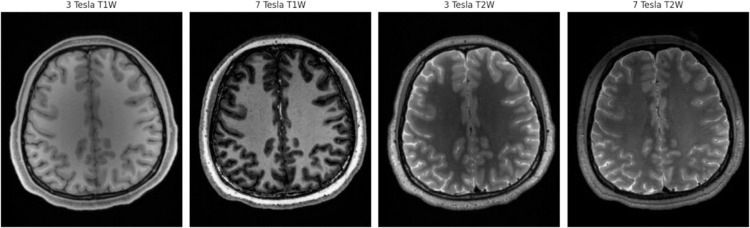
Different MRI scan contrasts and strengths provided by the paired dataset [15].

The dataset provides both volumes with the original characteristics and aligned versions in which the 3T T1w images were linearly registered to the 7T T1w images. Linear registration was performed via rigid-body alignment with translations and rotations, without scaling or nonlinear deformation, using FSL FLIRT to ensure anatomical correspondence between the 3T and 7T scans [15]. Due to model architecture considerations, and to make direct comparison of synthesized samples easier, we utilized the aligned versions in this study. Thus, all volumes contain 256 sagittal, 304 coronal, and 308 transverse slices. Fig 1 shows the different scan types provided by the paired dataset. Aside from linear registration and facial information removal from all MRI scans, the dataset utilized for this study had no other preprocessing applied.

For our semi-supervised approach, we employ an additional dataset of 20 7T volumes presented by Li *et al*. [28]. The dataset contains T1-weighted whole-brain scans from 20 healthy university volunteers (10 males, 10 females) aged 18–25 years, collected at the Beijing MRI Center for Brain Research. Scans were acquired on the same day using an investigational Nova 7T scanner. The 7T protocol included a high-resolution 3D T2-weighted SPACE sequence (0.4 × 0.4 × 1 mm^3^) and an isotropic 0.7 mm^3^ T1-weighted MPRAGE sequence, both scanned along the hippocampal long axis and the AC-PC line. We only utilize the 7T T1-weighted data for our semi-supervised approach since we focus only on high-field synthesis of T1-weighted samples. Each 7T volume contains an acquisition matrix of size 320 × 320 × 256, resulting in a total of 11340 additional 64 x 64 x 64 patches to leverage for our semi-supervised learning approach.

All participants in the Chen *et al*. [15] and Li *et al*. [28] datasets provided informed written consent for data collection and sharing. The data are fully anonymized, and no identifiable personal information was used in this study. The original studies that collected these data were approved by their respective institutional review boards (IRBs) at their hosting institutions. As we only used anonymized, previously collected MRI data without any personal identifiers or interaction with subjects, additional IRB approval was not required at Florida Atlantic University. The datasets were accessed on February 15, 2025, and at no point did the authors have access to information that could identify individual participants. All data use complies with institutional and journal ethical standards.

### Data pre-processing

**Fig 2:**
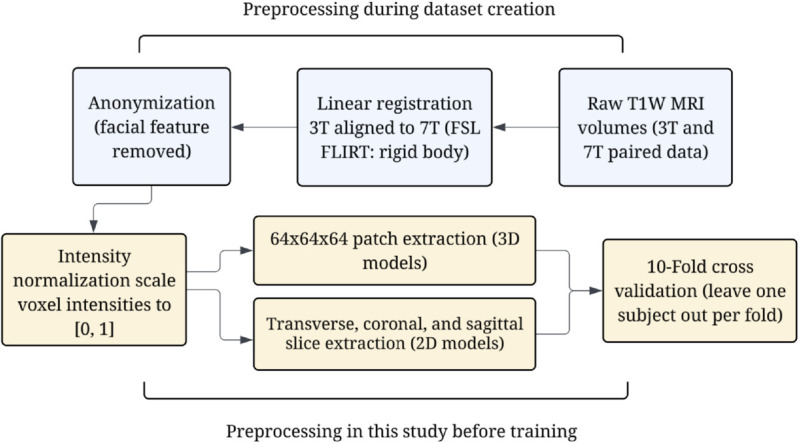
Dataset preprocessing workflow for paired T1W 7T and 3T MRI data [15].

For this study, each MRI volume was normalized to a range of [0,1] before training/testing, ensuring standardization while maintaining subject level intensity distributions. We utilize a 10-fold cross validation technique in which one full MRI volume is used for testing, while the remaining 9 are used for training, following the leave one out cross validation approach utilized by previous studies [22,25,30]. Next, for the patch-based method, each of the volumes were split into 64 x 64 x 64 patches with an overlapping stride of 32. This resulted in 567 patches per volume, for a total of 5103 paired patches in the training set. For the slice-by-slice synthesis methods, we extracted all paired slices for each direction, resulting in 8,800 paired slices for training and testing. Once again, we utilized 10-fold cross validation and set aside a full MRI volume for testing during each fold. Fig 2 illustrates the overall preprocessing workflow that we used for this study.

### 3D U-Net based models

**Fig 3:**
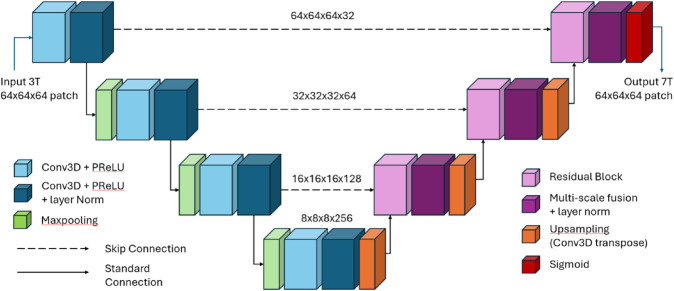
3D multi-scale fusion residual U-Net (3D FR-U-Net) architecture.

To address the challenge of volumetric medical image translation, we propose a 3D U-Net inspired architecture designed for learning fine-grained structural features while maintaining global spatial consistency. The architecture follows an encoder-decoder paradigm with symmetric skip connections, allowing multi-scale feature integration and efficient high-resolution 3D image reconstruction. Fig 3 illustrates the overall architecture of our 3D Multi-Scale Fusion Residual U-Net. Our architecture is based on the U-Net presented by Li *et al*. [28], with key modifications such as attention through residual blocks and multi-scale fusion.

The encoder consists of four hierarchical levels, each composed of 3D convolutional layers with parametric rectified linear unit (PReLU) activations, followed by layer normalization for stable gradient propagation. A max-pooling operation with a stride of (2 × 2 × 2) is applied at each level to downsample spatial resolution while increasing feature dimensionality. The encoder stages are as follows:

1. 3D convolutional layers with 32 feature channels; spatial resolution 64 × 64 × 64
2. 3D convolutional layers with 64 feature channels; spatial resolution 32 × 32 × 32
3. 3D convolutional layers with 128 feature channels; spatial resolution 16 × 16 × 16
4. (Bottleneck) 3D convolutional layers with 256 feature channels; spatial resolution 8 × 8 × 8

The decoder reconstructs the volumetric MRI using 3D transposed convolutions (deconvolutions) with residual blocks and multi-scale fusion. The transposed convolutions has a stride of (2 × 2 × 2) at each level to up sample spatial resolution while reducing feature dimensionality. The decoder stages are as follows:

1. Transposed convolution (128 channels) and concatenation with encoder level 3
2. Transposed convolution (64 channels) and concatenation with encoder level 2
3. Transposed convolution (32 channels) and concatenation with encoder level 1
4. Final Reconstruction: 1×1×1 convolution followed by a sigmoid activation function

Residual blocks are introduced at each decoding stage to refine feature maps [31], ensuring better anatomical accuracy. Each residual block consists of two (3 × 3 × 3) convolutional layers with PReLU activations, and a skip connection that adds the input directly to the output. To capture fine details across different receptive fields, we integrate a multi-scale feature extraction strategy [31]. At each decoder stage, three parallel 3D convolutional branches process the input feature maps using (1 × 1 × 1), (3 × 3 × 3), and (5 × 5 × 5) kernels, followed by channel-wise concatenation.

To optimize training, we employ a hybrid loss function that combines Mean Squared Error (MSE) for intensity-based reconstruction and Structural Similarity Index Measure (SSIM) to enforce perceptual similarity. The hybrid loss function is defined by Eq 1, where *λ* is a weighting factor that controls the influence of SSIM loss. We set *λ* = 0.7 to emphasize perceptual quality while maintaining voxel intensity consistency.

$$Hybrid\;Loss=\underset{{ℒ}_{\text{MSE}}}{\underbrace{MSE\;Loss}}+\lambda \times \underset{{ℒ}_{\text{SSIM}}}{\underbrace{SSIM\;Loss}}$$ (1)

Eq 4 defines the Structural Similarity Index Measure (SSIM) between an image and a target image, where $\mu_{y}$ and $\mu_{\hat{y}}$ represent the mean voxel intensities, $\sigma_{y}^{2}$ and $\sigma_{\hat{y}}^{2}$ denote the variances, $\sigma_{y\hat{y}}$ is the covariance, and *C*_1_ and *C*_2_ are stabilizing constants. SSIM loss, shown in Eq 2, calculates the mean SSIM score across all *N* voxel-wise patches or slices. In contrast, mean squared error (MSE) loss, shown in Eq 3, computes the average squared difference between the predicted voxel intensities ${\hat{y}}_{i}$ and the ground truth voxel intensities *y*_*i*_ over *N* samples.

$$SSIM\;Loss=1-\frac{1}{N}\sum_{i=1}^{N}SSIM(y_{i},{\hat{y}}_{i})$$ (2)

$$MSE\;Loss=\frac{1}{N}\sum_{i=1}^{N}(y_{i}-{\hat{y}}_{i}{)}^{2}$$ (3)

$$SSIM(y,\hat{y})=\frac{\left(2\mu_{y}\mu_{\hat{y}}+C_{1})(2\sigma_{y\hat{y}}+C_{2}\right)}{\left(\mu_{y}^{2}+\mu_{\hat{y}}^{2}+C_{1})(\sigma_{y}^{2}+\sigma_{\hat{y}}^{2}+C_{2}\right)}$$ (4)

The model is trained using the Adam optimizer with a learning rate of $2\;\times \;10^{-5}$. A batch size of 8 is used due to memory constraints, and training is conducted for 100 epochs. To prevent overfitting, early stopping is applied based on the validation loss, with a patience of 10 epochs.

### Model ablation

To evaluate the effect of each architectural modification on synthesis performance, we conducted an ablation study based on a standard 3D U-Net. Starting with the baseline model, we incrementally introduced the following components: (1) reduced network depth by one layer to mitigate overfitting, (2) a hybrid loss function combining SSIM and MSE, (3) residual blocks and multi-scale fusion (MSF) modules in the decoder, and (4) semi-supervised training. Each addition was assessed to isolate its individual contribution to the overall performance.

### Semi-supervised training

In this study, we implemented a semi-supervised learning framework to enhance the translation of synthetic 3T MRI patches to high field 7T MRI. Our approach leverages both paired (3T → 7T) data for supervised learning and unpaired 7T MRI patches to enforce consistency constraints on the model’s predictions. Specifically, we utilized the 3D Multi-Scale Fusion Residual U-Net as our backbone model as presented in the previous sections, trained using a hybrid loss function that combines Mean Squared Error (MSE) and Structural Similarity Index (SSIM) for paired data. To incorporate the unpaired 7T data, we introduced a consistency loss, which encourages the network to produce structurally coherent outputs by minimizing L1 and SSIM differences between predicted and real 7T images.

The consistency loss for unpaired 7T MRI patches is defined by Eq 5, where x7T represents the real unpaired 7T MRI patch, f(x7T) is the corresponding output generated by the model, and SSIM measures the structural similarity between the input and output. The hyperparameter *α* controls the contribution of SSIM loss to ensure structural coherence.

$${ℒ}_{\text{consistency}}={𝔼}_{x_{\tau T}}\left[\|x_{\tau T}-f(x_{\tau T}){\|}_{1}+\alpha \left(1-SSIM(x_{\tau T},f(x_{\tau T}))\right)\right]$$ (5)

The total loss function used for training is defined in Eq 6, where $L_{\text{supervised}}$ denotes the hybrid loss computed for paired (3T→7T) samples, and *λ* is a weighting factor that controls the influence of the consistency loss during training.

$${ℒ}_{\text{total}}={ℒ}_{\text{supervised}}+\lambda {ℒ}_{\text{consistency}}$$ (6)

**Fig 4:**
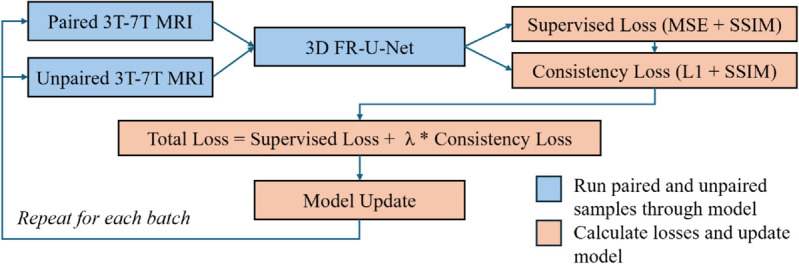
Semi-supervised model workflow.

The dataset was structured to maintain a 1:1 ratio of unpaired to paired samples, ensuring effective regularization without over-constraining the model. Fig 4 shows the workflow of the semi-supervised training process. During each batch, the loss was individually calculated for the paired and unpaired samples after a forward pass through the model, resulting in a combined loss which was used to update the model. This was repeated until early stopping convergence with patience of 10 epochs was achieved or 100 epochs total. Training was conducted using a custom TensorFlow implementation with a subclassed Keras model, where the semi-supervised optimization was performed via a modified ‘train step()‘ function to jointly compute gradients from both paired and unpaired loss components. Model evaluation was performed exclusively on the supervised validation dataset, ensuring that performance comparisons remained consistent with fully supervised baselines.

### Slice based methods

As a benchmark synthesis method, we use the pix2pix conditional GAN architecture extended with ResNet blocks to introduce elements of attention. Pix2pix has been widely used for medical image synthesis, including MRI synthesis, and is utilized as a benchmark GAN by many studies [9,11,12]. We follow the implementation details presented by [32], only modifying the generator architecture to add 3 ResNet blocks between the down sampling and up sampling layers. We did this due to introduce a basic form of attention, which we also utilized in the 3D volumetric models and allowed us to achieve an improved baseline performance.

We selected pix2pix as a baseline due to its widespread adoption in medical image synthesis literature especially in data-constrained scenarios. While more advanced models such as DDPMs and transformer-based architectures exist, they typically require large datasets and extensive tuning. These models were excluded due to incompatibility with our small-data setting and the unavailability of pretrained implementations suitable for volumetric MRI synthesis.

### Quantitative evaluation metrics

For our evaluation metrics, we used three main qualitative metrics, structural similarity index measure (SSIM), peak signal-to-noise ratio (PSNR), and normalized mean squared error (NMSE), which are commonly used in conditional image synthesis tasks. Eq 4, Eq 7, and Eq 8 represent PSNR, NMSE, and SSIM respectively.

$$PSNR=10\log_{10}\left(\frac{\max^{2}(y(x),G(x))}{\frac{1}{N}\sum \|y(x)-G(x){\|}_{2}^{2}}\right)$$ (7)

$$NMSE=\frac{\sum_{i=1}^{n}(f_{i}-y_{i}{)}^{2}}{\sum_{i=1}^{n}(y_{i}{)}^{2}}$$ (8)

In the PSNR Eq 7, y(x) represents the ground truth image, while G(x) is the reconstructed image. The numerator corresponds to the maximum possible pixel value, while the denominator contains the Mean Squared Error (MSE), which quantifies the difference between the two images. The NMSE Eq 8 normalizes the squared error between y(x) and G(x) by the squared norm of the ground truth image, providing a scale-invariant measure of error. The SSIM Eq 4 evaluates structural similarity by considering luminance, contrast, and structural details between the images. It incorporates mean intensity, variance, and covariance terms to measure perceptual quality while stabilizing computations with predefined constants.

To gain a more comprehensive understanding of the synthesis quality of the MRI volume, we report the above metrics separately for each slice orientation of the synthetic MRI samples. Since MRI volumes can be viewed from multiple axes, each providing a distinct perspective on anatomical structures, evaluating synthesis quality across all orientations allows us to identify any discrepancies. This analysis helps determine if certain orientations exhibit lower synthesis fidelity.

### Qualitative evaluation metrics

Aside from standard qualitative metrics, we incorporate segmentation-based volumetric evaluation to assess the structural fidelity of the synthesized 7T MRI volumes. Specifically, we utilize VolBrain [19], a high-resolution neuroimaging segmentation pipeline known for its anatomical precision in fine-grained whole brain structure delineation. VolBrain is based on AssemblyNet, a large ensemble of 3D convolutional neural networks (CNNs) trained to segment 132 brain structures at high resolution using a multi-scale, two-assembly U-Net architecture [19]. Table 2 describes the regional volumetric areas of interest analyzed using VolBrain, which serve as key reference points for our assessment. We assess anatomical consistency based on medically established associations between structural changes in each region and specific neurological or psychiatric conditions [33–36].

**Table 2:** Volumetric segmentation masks and clinical relevance.

Region	Description	Clinical / Research Relevance
White Matter (WM)	Bundles of myelinated axons connecting different brain regions.	Vital for signal transmission; changes seen in MS, aging, trauma.
Grey Matter (GM)	Neuronal cell bodies, dendrites, and synapses.	Crucial for cognition; atrophy linked to Alzheimer’s, epilepsy.
Cerebrospinal Fluid (CSF)	Fluid in ventricles and subarachnoid space.	Changes in CSF volume may indicate hydrocephalus, atrophy.
Brain (WM+GM)	Combined brain tissue volume excluding CSF.	Global measure of brain health or atrophy.
Intracranial Cavity (IC)	Total volume inside the skull, includes brain + CSF.	Normalization reference for relative volume calculations.
Cerebrum	Largest brain portion including cortex and subcortex.	Overall brain bulk; major cognitive and motor functions.
Cerebellum	Coordinates voluntary movement, posture, and balance.	Sensitive to degeneration (e.g., ataxias).
Brainstem	Connects brain and spinal cord; controls autonomic functions.	Involved in life-sustaining processes.
Hippocampus	Key structure for memory and learning.	Biomarker for Alzheimer’s and epilepsy.
Thalamus	Relay center for sensory and motor signals to the cortex.	Involved in consciousness and alertness.
Caudate	Part of the basal ganglia; involved in learning and motor control.	Altered in Huntington’s and Parkinson’s disease.
Putamen	Motor control, part of the basal ganglia circuit.	Affected in movement disorders.
Amygdala	Emotion processing, fear response.	Implicated in PTSD, anxiety, depression.
Frontal Lobe	Executive functions, decision making, voluntary movement.	Target of study in TBI, schizophrenia, ADHD.
Temporal Lobe	Memory, auditory processing, language.	Degeneration common in dementia.
Parietal Lobe	Sensory integration and spatial reasoning.	Involved in neglect syndromes, stroke.
Occipital Lobe	Visual processing.	Can show lesions in MS, tumors.
Lateral Ventricles	Largest CSF-filled spaces in the brain.	Enlargement may indicate atrophy or hydrocephalus.
3rd and 4th Ventricles	Midline CSF compartments.	Altered size can reflect intracranial pressure changes.
External CSF	CSF in subarachnoid space outside the brain.	Increases with brain atrophy or trauma.

Clinical/research relevance statements are supported by prior literature as cited in the text above the Table [33–36]. In the context of T1w-only volumetric synthesis, standalone diagnostic or research value is most applicable to global brain metrics (WM, GM, WM+GM, IC, CSF), lobar volumes, basal ganglia volumes (caudate, putamen), cerebellar volume, and ventricular size. Other regions, such as hippocampus, amygdala, thalamus, and fine subcortical structures, are typically interpreted in conjunction with additional 7T contrasts (T2w, T2*w, SWI) or functional/spectroscopic imaging to capture physiological or microstructural changes not visible in T1w volumetry.

By comparing segmentation outputs between real and synthetic 7T MRIs, we can quantify structural differences and assess the model’s ability to preserve detailed anatomical features. VolBrain performs voxel-wise segmentation across an anatomical hierarchy, providing a comprehensive breakdown of each MRI volume into labeled subregions. We utilize these anatomical segmentations to quantify the discrepancy between ground truth and synthetic outputs, capturing volumetric deviations across specific regions. We compute segmentation-derived metrics, including the Dice Similarity Coefficient (DSC) and Hausdorff Distance (HD), to measure overlap accuracy and boundary precision between real and synthetic 7T segmentations. DSC and HD are widely used to assess segmentation performance in medical imaging [28]. DSC quantifies how similar two sets are by comparing the number of overlapping voxels to the total number of voxels in both segmentations, as described by Eq 9.

$$DSC=\frac{2\times |A\cap B|}{\left|A|+|B\right|}$$ (9)

The Hausdorff Distance (HD) is a boundary-based metric used to quantify the spatial discrepancy between two segmentation outputs. It measures the maximum distance from a point in one segmentation to the closest point in the other. Formally, given two sets of points A and B, the directed Hausdorff distance from A to B is defined by Eq 10, and the symmetric Hausdorff Distance is then calculated as shown in Eq 11.

$$h(A,B)=\max_{a\in A}\min_{b\in B}\|a-b\|$$ (10)

H(A,B)=max(h(A,B), h(B,A)) (11)

This metric captures the worst-case scenario of mismatch between boundaries, making it sensitive to outliers. Therefore, we will use the 95th percentile Hausdorff Distance (HD95) to provide a more robust assessment by excluding extreme outlier deviations.

## Results

This section presents the results of the experiments outlined in the Methods section, focusing on the evaluation of synthesized 7T MRI volumes. The results are analyzed using quantitative metrics such as PSNR, NMSE, and SSIM, as well as segmentation-derived evaluations including Dice Similarity Coefficient (DSC) and Hausdorff Distance. Additionally, qualitative visual comparisons provide further insights into the anatomical fidelity of the generated images.

### Synthesis quality

Table 3 presents the leave-one-out performance by slice orientation from the ablation study on the components of our final semi-supervised 3D FR-U-Net architecture. Subject 8 was used for validation, while the remaining 9 subjects were used for training. As shown, each architectural modification progressively improves structural similarity and other performance metrics, validating the contributions of each component.

**Table 3:** Ablation study of the semi-supervised 3D FR-U-Net architecture.

Additional Components	Transverse			Coronal			Sagittal		
	PSNR	SSIM	NMSE	PSNR	SSIM	NMSE	PSNR	SSIM	NMSE
Standard 3D U-Net	24.98	0.7248	0.2710	**22.96**	0.7212	**0.1682**	**23.40**	0.7153	**0.2208**
Hybrid Loss + Depth	**25.07**	0.7335	0.2545	22.77	0.7309	0.1750	23.25	0.7286	0.2333
MSF + Residual Block	24.48	0.7404	0.3256	22.23	0.7370	0.2015	22.89	0.7373	0.2347
Semi-supervised FR-U-Net	24.76	**0.7459**	**0.2534**	22.20	**0.7423**	0.2044	22.91	**0.7414**	0.2302

Performance is reported in terms of PSNR, SSIM, and NMSE across three anatomical planes for each network variant. Each row adds one or more components to the base 3D U-Net, showing the incremental performance improvements by slice orientation using PSNR, SSIM, and NMSE.

Table 4 shows the 10-fold cross-validation results by slice orientation for the three generative models evaluated in this study. The best-performing metrics for each orientation are highlighted in bold. Among the models tested, the semi supervised 3D FR-U-Net achieved the highest overall in SSIM metrics.

**Table 4:** Mean ± standard deviation of PSNR, SSIM, and NMSE by slice orientation for different generative models.

Method	Transverse			Coronal			Sagittal		
	PSNR	SSIM	NMSE	PSNR	SSIM	NMSE	PSNR	SSIM	NMSE
Pix2Pix with Attention	21.494 ± 1.4866	0.6547 ± 0.02348	0.6106 ± 0.30916	20.681 ± 1.1778	0.6533 ± 0.02358	0.4284 ± 0.26167	20.388 ± 1.3153	0.6573 ± 0.02382	0.4286 ± 0.20523
FR-U-Net	23.173 ± 1.6355	0.7339 ± 0.02794	0.3621 ± 0.15251	**22.489 ± 1.6881**	0.7367 ± 0.02708	**0.2270 ± 0.03479**	22.025 ± 1.5405	0.7364 ± 0.02849	0.2478 ± 0.05068
Semi-supervised FR-U-Net	**23.254 ± 1.7516**	**0.7368 ± 0.02731**	**0.3616 ± 0.21571**	22.476 ± 1.6832	**0.7396 ± 0.02698**	0.2391 ± 0.06695	**22.051 ± 1.5276**	**0.7391 ± 0.02822**	**0.2379 ± 0.04096**

Performance is reported as mean ± standard deviation of each metric across slices by orientation for each generative model.

To assess the effectiveness of our semi-supervised 3D FR-U-Net, we conducted paired t-tests comparing its performance to two baselines: Pix2Pix and a fully supervised 3D FR-U-Net. The semi-supervised 3D FR-U-Net significantly outperformed Pix2Pix in all slice orientations for SSIM and PSNR (all p < 0.0001), and also achieved significantly lower NMSE in the transverse (p = 0.0228) and sagittal (p = 0.0135) planes, with coronal NMSE showing a non-significant trend toward improvement (p = 0.066). In contrast, no statistically significant differences were found when comparing the semi-supervised FR-U-Net with the supervised FR-U-Net, across any metric or orientation (all p > 0.28). The corresponding t-values for each comparison are summarized in Table 5.

**Table 5:** Paired t-test statistics (t-values) comparing the semi-supervised FR-U-Net to baseline models across image quality metrics and slice orientations.

Models Compared	Transverse			Coronal			Sagittal		
	SSIM	PSNR	NMSE	SSIM	PSNR	NMSE	SSIM	PSNR	NMSE
*semi-supervised 3D FR-U-Net vs. Pix2Pix*	**13.624**	**6.712**	**-2.743**	**13.577**	**6.589**	**-2.091**	**13.149**	**7.353**	**-3.064**
*semi-supervised 3D FR-U-Net vs. 3D FR-U-Net*	-1.148	-0.604	0.019	-1.124	0.118	-0.957	-1.040	-0.197	0.905

### Anatomical segmentation

**Fig 5:**
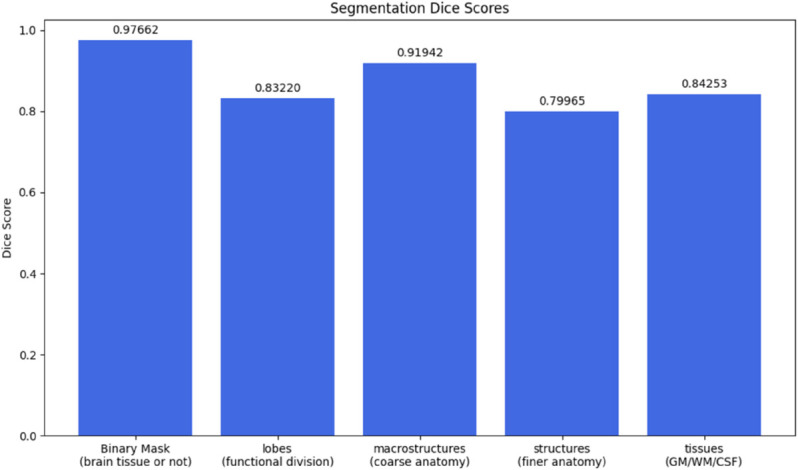
Segmentation Dice scores across anatomical categories for synthetic T1W 7T volumes. Segmentation dice scores are reported as average across all 10-fold test volumes from the paired dataset by Chen *et al*. [15]. Dice similarity coefficients are shown for five segmentation classes using VolBrain: (1) binary brain mask (brain tissue vs. non-tissue), (2) lobar segmentation based on functional divisions, (3) macrostructures representing coarse anatomical groupings, (4) finer anatomical structures, and (5) primary tissue classes.

**Fig 6:**
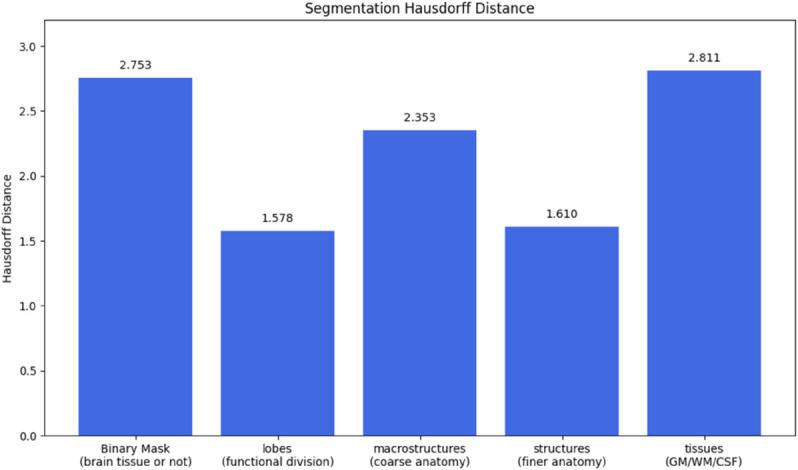
Hausdorff distances across anatomical segmentation categories for synthetic 7T volumes. Lower values indicate better anatomical boundary alignment between synthetic and real segmentations.

Fig 5 shows the average Dice Scores by segmentation map types between the ground truth T1W 7T and synthetic T1W 7T MRI volumes. VolBrain produces 5 different segmentation masks, including a binary mask with 1 class, a lobes mask with 12 classes, a macrostructure mask with 6 classes, a structures class with 132 classes, and a tissues mask with 7 classes. Fig 6 shows the average Hausdorff-95 distance between the ground truth and synthetic 7T MRI volumes for the same 5 segmentation mask types.

To contextualize the segmentation results from synthetic 7T images, we first quantified the volumetric deviations between 3T-derived segmentations and those obtained from the 7T scans (Tables 6 and 7). This comparison is not intended to establish absolute accuracy as both 3T and 7T acquisitions are genuine ground-truth images, but rather to evaluate the degree of volumetric consistency each has with 7T-derived segmentations. Across the 21 clinically relevant regions examined, synthetic 7T volumes showed a deviation profile that was generally comparable to, and in several smaller subcortical structures closer to, the 7T-derived volumes than those obtained directly from 3T images. If, as suggested in prior literature, ultra-high-field 7T imaging provides more precise volumetric estimates due to its higher resolution and contrast, then these findings represent a clear support for the proposed synthesis method, demonstrating that synthetic 7T images can help bridge the gap between standard 3T acquisitions and the volumetric fidelity associated with 7T imaging.

**Table 6:** Comparison of ground truth 3T and 7T volumetric analysis.

Region	Ground Truth 7T		Ground Truth 3T		% Difference
	cm^3^	%	cm^3^	%	
**Global Brain Metrics**					
White Matter (WM)	409.262	30.2322	459.393	33.1943	-10.912
Grey Matter (GM)	690.165	50.9096	746.664	53.9261	-7.567
Brain (WM+GM)	1099.429	81.1418	1206.056	87.1204	-8.841
Intracranial Cavity (IC)	1355.201	100.0000	1385.661	100.0000	-2.198
Cerebrospinal Fluid (CSF)	239.683	17.6650	162.003	11.6087	**47.950**
**Macrostructure Segmentation**					
Cerebrum	987.407	72.8668	1073.781	77.5369	-8.044
Cerebellum	102.823	7.5922	121.333	8.7916	**-15.256**
Brainstem	16.091	1.1931	17.602	1.2711	-8.584
**Subcortical Structures**					
Hippocampus	6.053	0.4535	6.995	0.5092	-13.467
Thalamus	16.322	1.2079	16.598	1.1997	-1.663
Caudate	5.804	0.4285	6.398	0.4624	-9.284
Putamen	9.382	0.6952	9.153	0.6623	2.502
Amygdala	1.570	0.1163	2.223	0.1606	**-29.375**
**Cortical Regions**					
Frontal Lobe	180.514	13.3334	192.400	13.8908	-6.178
Temporal Lobe	115.179	7.9411	127.696	8.7373	**-9.802**
Parietal Lobe	107.091	7.9125	116.270	8.3949	-7.895
Occipital Lobe	86.287	6.3851	87.368	6.3162	-1.237
**Ventricular & CSF Measurements**					
Lateral Ventricles (Total)	18.271	1.3136	16.703	1.1731	9.388
3rd Ventricle Volume	0.969	0.0715	0.684	0.0496	41.667
4th Ventricle Volume	1.472	0.1104	1.390	0.1017	5.899
External CSF Volume	217.698	16.0759	142.376	10.2236	**52.904**

Each entry reports volume (in cm^3^) and percentage of total intracranial cavity, comparing ground truth 7T (GT) with the ground truth 3T volumes that are used as input for synthetic image translation. Percentage difference is calculated as: %difference = 100 × (3T - 7T) / 7T. The largest %difference in each category is highlighted in bold.

**Table 7:** Synthesis volume by brain region and difference between ground truth and synthetic sample.

Region	Ground Truth 7T		Synthetic Sample 7T		% Error
	cm^3^	%	cm^3^	%	
**Global Brain Metrics**					
White Matter (WM)	409.262	30.2322	415.564	30.2830	1.540
Grey Matter (GM)	690.165	50.9096	683.018	49.5413	-1.036
Brain (WM+GM)	1099.429	81.1418	1125.340	82.0601	2.357
Intracranial Cavity (IC)	1355.201	100.0000	1370.616	100.0000	1.137
Cerebrospinal Fluid (CSF)	239.683	17.6650	228.795	16.7366	**-4.543**
**Macrostructure Segmentation**					
Cerebrum	987.407	72.8668	1002.440	73.0755	1.522
Cerebellum	102.823	7.5922	112.559	8.2295	**9.469**
Brainstem	16.091	1.1931	16.480	1.2032	2.418
**Subcortical Structures**					
Hippocampus	6.053	0.4535	6.228	0.4583	2.891
Thalamus	16.322	1.2079	16.071	1.1742	-1.538
Caudate	5.804	0.4285	5.752	0.4196	-0.896
Putamen	9.382	0.6952	9.040	0.6612	-3.645
Amygdala	1.570	0.1163	1.650	0.1202	**5.096**
**Cortical Regions**					
Frontal Lobe	180.514	13.3334	184.646	13.4645	2.289
Temporal Lobe	115.179	7.9411	113.951	8.2910	-1.066
Parietal Lobe	107.091	7.9125	109.554	7.9815	**2.300**
Occipital Lobe	86.287	6.3851	85.925	6.2750	-0.420
**Ventricular & CSF Measurements**					
Lateral Ventricles (Total)	18.271	1.3136	18.531	1.3178	1.423
3rd Ventricle Volume	0.969	0.0715	0.907	0.0662	**-6.398**
4th Ventricle Volume	1.472	0.1104	1.448	0.1071	-1.630
External CSF Volume	217.698	16.0759	206.505	15.1446	-5.142

Each entry reports volume (in cm^3^) and percentage of total intracranial cavity, comparing ground truth (GT) with synthetic samples. Percentage error is calculated as: %Error = 100 × (Synthetic - GT) / GT. The largest %error in each category is highlighted in bold.

Table 7 shows the average volume across all 10-fold test volumes for both the synthetic and GT 7T MRI. VolBrain provides 132 different segmentation classes, but for this study we focus on a select 21 key structures of clinical relevance that we organize into 5 distinct groups. Table 2 from the methods section describes the clinical relevance of each of the structures, highlighting the potential implications and synthesis fidelity of certain clinically useful structures.

Table 8 shows the average asymmetry between select brain structures, including the hippocampus, caudate, thalamus, and lateral ventricles. The error between the ground truth and synthetic samples is also indicated.

**Table 8:** Synthesis volume by brain region and difference between ground truth and synthetic sample asymmetry indices.

Asymmetry Indices	Ground Truth	Synthetic Samples	% Error
Hippocampal Asymmetry	-4.7443	-1.8469	**-61.0712**
Caudate Asymmetry	3.6747	3.8648	5.1746
Thalamic Asymmetry	0.6119	0.5088	-16.8543
Lateral Ventricles Asymmetry	5.7013	5.5633	-2.4215

Asymmetry indices are calculated using the formula: % Error = 100 × (Synthetic - GT) / GT, comparing left and right volumes of selected brain structures. The largest %difference is highlighted in bold.

**Fig 7:**
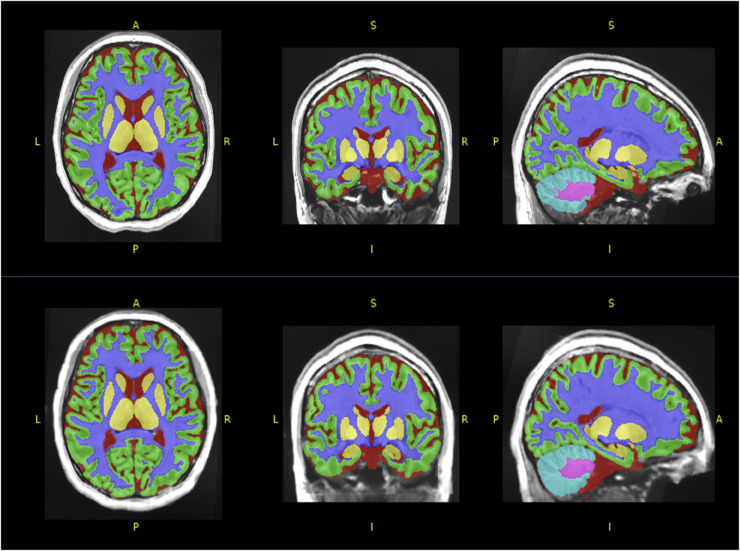
Segmentation map comparison of subject 2 ground truth 7T (top) and synthetic 7T (bottom).

Finally, Fig 7 plots the segmentation mask overlayed on the ground truth and synthetic samples. The figure highlights the differences indicated by the structural data in Table 7, with the synthetic sample having increased white matter due to less clear boundaries between gray and white matter structures. Additionally, fine anatomical details and boundaries are smoothed out, as shown by the differences in brain cerebellum structural boundaries, shown by the pink structure.

### Qualitative assessment

**Fig 8:**
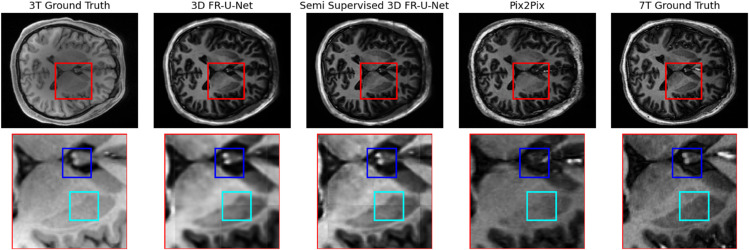
Comparison of subject 8 synthetic 7T and ground truth 7T and 3T T1W images.

**Fig 9:**
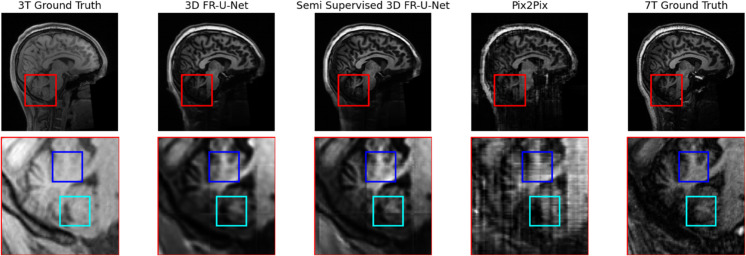
Cerebellum comparison of subject 8 synthetic 7T and ground truth 7T and 3T T1W images.

Finally, we present a qualitative analysis of the synthetic image regions. As shown in Fig 8, while the synthetic images faithfully capture the overall structure of gray and white matter, they struggle to preserve finer anatomical details with the same level of sharpness. High-contrast regions—particularly bright features absent in the original 3T scans, such as the area highlighted by the box—are consistently lost across all synthetic methods. However, compared to the original 3T input images, some anatomical regions show noticeable improvements in detail and resolution. Fig 9 illustrates a slice of the cerebellum in the original 3T, ground truth 7T, and synthetic MRI outputs. The areas highlighted by the blue boxes in the synthetic images exhibit enhanced anatomical detail compared to the 3T input, closely matching the 7T ground truth. This demonstrates how synthetic imagery can serve as a useful alternative high-field view for specific anatomical regions.

The outputs from the FR-U-Net and semi supervised FR-U-Net models appear visually similar; however, the semi-supervised variant demonstrates improved sharpness and clarity in fine-detail regions. In contrast, the pix2pix baseline produces sharper-looking images overall, but introduces clear synthesis artifacts and omits important anatomical structures entirely.

3T and 7T acquisitions have distinct artifact profiles, with B1 field inhomogeneity being a known factor at 7T. In our dataset, such artifacts are present in the ground truth 7T images and therefore could be learned by the model during training. While our results suggest that the network preserved large-scale anatomical structures and contrast, it is possible that certain 7T-specific artifacts are also reproduced in the synthetic images. These qualitative patterns are consistent with our quantitative findings, where higher volumetric and boundary errors (Tables 6 and 7) occurred in small, high-curvature regions compared to larger cortical areas. At present, our method does not explicitly remove such artifacts. However, future work could incorporate artifact-robust training strategies, such as artifact simulation and augmentation, or adversarial discriminators trained to penalize known artifact patterns, to reduce their influence.

## Discussion

This study presents a semi-supervised 3D multi-scale fusion residual U-Net (semi-supervised 3D FR-U-Net) for synthesizing high-resolution 7T MRI volumes from 3T inputs, with a novel evaluation framework centered on anatomical consistency. While the model demonstrated improved performance across standard synthesis metrics such as SSIM, PSNR, and NMSE, we argue that traditional pixel-level metrics are insufficient for determining real-world utility. To help bridge this gap, we introduced a segmentation-based evaluation pipeline using VolBrain, which allowed for interpretable, structure-specific analysis of anatomical fidelity across synthesized images.

Our model achieved the highest quantitative performance across all slice orientations compared to both fully supervised 3D U-Nets and a 2D slice-based pix2pix baseline. Most notably, it outperformed prior work by Siam *et al*. [18], who reported an SSIM of 71.07% and a PSNR of 18.62 using a CycleGAN-based method with data augmentation. Even in the transverse orientation, our model achieved a PSNR of 23.254 and SSIM of 73.682%. Although Siam *et al*. were able to increase SSIM above 80%, this required further preprocessing through skull stripping. Our methodology highlights the practicality of our approach in real-world clinical workflows, where extensive preprocessing may be undesirable or infeasible.

While our method demonstrates consistent improvements in SSIM, PSNR, and NMSE over existing baselines, we acknowledge that these gains are modest in absolute terms. This is not unexpected given the difficulty of synthesizing high-resolution, ultra-high-field MRI from a dataset of only 10 paired volumes, which is an extreme low-data regime by deep learning standards. In such settings, overfitting, noise amplification, and anatomical blurring are common challenges, and even marginal improvements can reflect meaningful architectural or learning-based advances. Moreover, traditional pixel-level metrics like SSIM and PSNR often undervalue perceptual and structural fidelity, particularly in clinically relevant regions. For example, sharper-looking images with slightly lower SSIM scores may yield more accurate anatomical segmentations or highlight diagnostically useful features. To address these limitations, our segmentation-based evaluation, focused on key substructures such as the hippocampus and cerebellum, offers a more informative view of region-specific strengths and weaknesses in anatomical preservation. Taken together, our results suggest that modest gains in conventional metrics, especially in low-data contexts, should be interpreted alongside structure-aware assessments that better reflect clinical relevance.

VolBrain analysis revealed that the model preserved major anatomical regions with high fidelity, achieving a Dice score of 0.9766 for whole-brain masks and greater than 0.84 for white and gray matter. Volumetric error was under 3% for most global and lobar structures, and asymmetry indices in regions such as the caudate and thalamus showed less than 6% deviation from ground truth. These results suggest the model could be clinically useful for tasks involving general brain morphology or basal ganglia analysis.

However, the same analysis also exposed important limitations. Despite relatively low volumetric error ( 2.9%) in the hippocampus, the asymmetry index deviated by more than 60%, indicating a substantial failure to preserve lateralized structural features. Given that inter-hemispheric hippocampal asymmetry is a critical marker in diagnosing Alzheimer’s disease and localizing seizure foci in epilepsy [7,37], this finding suggests that our model, in its current form, is not yet suitable for clinical applications involving the hippocampus or other fine subcortical structures. Hausdorff distance analysis further confirmed reduced boundary precision in small regions such as the amygdala and caudate compared to larger anatomical structures. Future studies should investigate fine-structure localized loss functions or the use of down-stream task guidance during training to help maintain fine grained anatomical fidelity.

These results reinforce the importance of the proposed segmentation-based evaluation framework. By moving beyond standard image similarity metrics and assessing anatomical fidelity in clinically meaningful regions, our approach can provide structural insight when radiologist review is infeasible—such as in large-scale studies or low-resource settings. While we did not have access to expert human annotators in this study, future work incorporating radiologist or neurologist input will be essential to define clinically acceptable thresholds for volumetric, boundary, and asymmetry errors. Such expert validation will be critical for aligning automated evaluation methods with diagnostic standards and improving the clinical readiness of synthesis models.

The study has several limitations. Most notably, it relies on a very small dataset of only 10 paired 3T–7T volumes, which limits generalizability across populations, acquisition protocols, and disease states. Although the semi-supervised framework allowed effective use of unpaired 7T data, larger and more diverse datasets will be necessary to assess robustness and performance under pathological conditions with abnormal structures. The network was trained on a fixed 3T–7T contrast mapping with specific acquisition parameters, which constrains its applicability across the wide range of MRI contrasts and protocols encountered in clinical practice. While the dataset also contains T2-weighted images, only T1-weighted images were used in this study to align with standard segmentation benchmarks such as those required by VolBrain and reduce modality-related variability. However, the architecture is contrast-agnostic and could be extended to T2-weighted and FLAIR images given appropriate paired datasets. These modalities provide complementary physiological information, such as sensitivity to edema, demyelination, and lesion load—not as prominent in T1-weighted imaging, and represent a promising direction for future work. This specificity reflects a broader limitation of current deep learning methods in MRI translation, where domain shift due to scanner settings, contrast weightings, and sequence variants can significantly impair performance.

Additionally, while we included a widely used baseline (pix2pix) and conducted detailed ablation studies to isolate the effects of key architectural components, comparisons to more recent state-of-the-art models (e.g., diffusion models, transformers) were not performed. This was due to either the lack of publicly available pretrained models for our problem setting or their incompatibility with small-volume paired datasets like ours. We acknowledge this as a limitation and have explicitly framed our evaluations in the context of low-data synthesis scenarios.

Future work should explore multi-modal synthesis to better reflect real-world neuroimaging protocols and develop models with contrast-adaptive or domain-generalizing capabilities. Creating large datasets with diverse acquisition parameters may greatly improve algorithm performance across real-world clinical imaging scenarios.

Future studies should also investigate region-specific learning strategies or model adaptations, such as incorporating transformer or diffusion-based modules tailored for low-data settings. Furthermore, integrating clinician-derived evaluation criteria—especially for high-impact regions like the hippocampus—will be essential for transitioning from research-grade synthesis to clinically actionable imaging. Incorporating segmentation-based loss guidance could further steer the network toward improved structural fidelity by explicitly reinforcing anatomical accuracy during training.

## Conclusion

Overall, our semi-supervised 3D synthesis approach offers strong performance and promising anatomical fidelity in many brain regions, even under small data constraints. VolBrain-based segmentation revealed that the model preserves overall brain structure and may support applications in certain contexts, such as evaluating caudate volume or global brain atrophy. However, significant limitations remain in regions like the hippocampus, underscoring the need for targeted improvements and radiologist-informed evaluation frameworks. Additionally, the current results must be considered in the context of potential domain shifts, scanner- and protocol-specific dependencies, and the lack of validation on pathological cases, any of which could limit their generalizability to broader clinical populations. These findings mark an important step toward clinically interpretable synthetic MRI and demonstrate how segmentation-based assessments can clarify the true readiness of generative models for real-world use.
